# Paying for home care out-of-pocket is common and costly across the income spectrum among older adults

**DOI:** 10.1093/haschl/qxae180

**Published:** 2025-01-16

**Authors:** Karen Shen, Yang Yang, Katherine A Ornstein, Regina A Shih, Jennifer M Reckrey

**Affiliations:** Department of Health Policy and Management, Johns Hopkins Bloomberg School of Public Health, Baltimore, MD 21205, United States; Department of Health Policy and Management, Johns Hopkins Bloomberg School of Public Health, Baltimore, MD 21205, United States; Center for Equity in Aging, Johns Hopkins School of Nursing, Baltimore, MD 21205, United States; Department of Epidemiology, Emory Rollins School of Public Health, Atlanta, GA 30322, United States; Icahn School of Medicine at Mount Sinai, New York, NY 10029, United States

**Keywords:** aging, home care, long-term care, financial burden

## Abstract

Many older adults with personal care needs rely on paid caregivers to remain in the community (“home care”). Those without Medicaid or private long-term-care insurance must pay out-of-pocket for care. We used the Health and Retirement Study to identify the prevalence and financial burden of paying for home care out-of-pocket in 2002–2018, by income and dementia status. Over 600 000 people with personal care needs paid out-of-pocket for home care in a given year, 45% of whom have dementia. The quantity and cost of this care were substantial for people with dementia in particular: 51% of those with dementia paying out-of-pocket for home care spent ≥$1000/month. While the probability of paying out-of-pocket for home care increased sharply with income, 52% of people paying out-of-pocket for home care had incomes below 200% of the federal poverty line; this group faced high financial burdens of care. Policies aimed at easing the financial burden of home care are essential, particularly for low-income individuals with dementia who experience the greatest financial burden.

## Introduction

**Figure 1.**
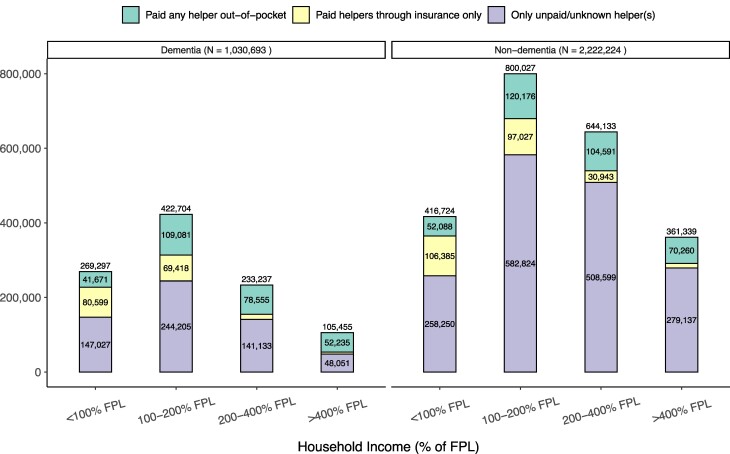
Annual average number of community-dwelling older adults with personal care needs receiving home care with different payment sources, by income and dementia status: 2002–2018 Health and Retirement Study (HRS). Based on a sample of 8181 respondent-years from HRS 2002–2018 (2797 dementia, 5384 non-dementia) who received help with at least 1 ADL and did not live in a nursing home or receive ADL help from their residence. Survey weights were used to compute the total number of people in each category, and then were divided by the number of years. The FPL in 2018 was $12 000 for an individual and $18 000 for a couple. Among all respondents aged 65+ years in our sample years, the income thresholds of 100%, 200%, and 400% FPL corresponded to the 12th, 42nd, and 74th percentiles, respectively. Abbreviations: ADL, activities of daily living; FPL, federal poverty line.

Many older adults, particularly those with dementia, rely on paid caregivers to meet their personal care needs at home (“home care”).^1-3^ Lack of comprehensive home care coverage in the United States due to exclusion of long-term care from Medicare, uneven coverage in Medicaid,^4-6^ and low take-up of private long-term-care insurance,^7^ means that many must pay out-of-pocket for home care, which can include privately hiring care through an agency or through the “gray market.”^8,9^ Using data from the Health and Retirement Study (2002–2018), we estimated that over 600 000 people annually with personal care needs paid out-of-pocket for home care, of whom 45% have dementia (Figure 1). Paying out-of-pocket for home care spans the income spectrum: over 52% of people paying out-of-pocket for home care had incomes below 200% of the federal poverty line.

Paying for home care out-of-pocket may constitute a substantial financial burden to older adults,^10^ particularly for people with dementia, who have both higher care needs^4^ and lower socioeconomic status^11^ than people without dementia. Prior work has described higher overall out-of-pocket health-related spending for people with dementia,^12,13^ but has not studied the contribution of home care spending specifically or examined how spending varies across people with different income levels. Studying out-of-pocket spending on home care specifically is important because, unlike many other out-of-pocket health care costs that may represent one-time payments or co-pays related to insurance-covered expenses, costs associated with home care are not covered by Medicare and typically represent long-term, recurring out-of-pocket costs. While Medicaid may cover home care for lower-income individuals, strict eligibility requirements and varying coverage of home care across state Medicaid programs may necessitate paying out-of-pocket for home care even among those with low incomes. Our study aimed to fill these gaps by describing the prevalence, cost, and financial burden of paying out-of-pocket for home care, by income and dementia status.

## Data and methods

Using the Health and Retirement Study, we included respondent-year observations in 2002–2018 where the respondent was 65 years or older and received help with at least 1 “personal care” activity (eating, bathing, toileting, dressing, getting into/out of bed, walking across a room) in the past month. We excluded people living in a nursing home or who received help through their residence. Our sample included 8181 respondent-years corresponding to 4584 unique individuals (a sample selection table is presented in Appendix Table S1).

Our primary outcome variable was whether respondents paid for any home care out-of-pocket. We defined this variable as respondents who had a paid helper meeting at least 1 of 2 conditions: (1) the helper was paid to help, but insurance did not help pay, or (2) insurance helped pay for the helper, but the respondent indicated that they paid a nonzero out-of-pocket payment for that helper (see Appendix Figure S1 for more details). Sensitivity analyses considered a more stringent definition of this variable where people who meet the first condition are also required to indicate that they paid a nonzero out-of-pocket payment. The remaining respondents were grouped into those who paid for home care using only insurance and those with only unpaid helpers or helpers with unknown payment source. We used this variable and survey weights to estimate the average size of the population paying out-of-pocket for home care across all years, and the prevalence of paying out-of-pocket for home care among people with personal care needs, by income and dementia status.

In order to further describe characteristics of people paying out-of-pocket for home care, we constructed a subsample of those paying out-of-pocket for home care who provided exact, non-missing hours and cost data (967 respondent-years, 772 unique individuals), corresponding to 81% of our sample paying out-of-pocket for home care (Appendix Figure S1). For this subsample, we described the following: (1) the hours per week from helpers paid out-of-pocket, (2) the total monthly out-of-pocket cost, and (3) the percentage of monthly income spent on home care (ie, “financial burden” of care^14^). We also considered an alternative definition of financial burden that expresses spending as a percentage of combined income and annuitized assets.^10^

We defined dementia using the 27-point Langa-Weir cognitive assessment score; people who score 6 or fewer are categorized as having dementia.^15^ To categorize income, we used total income for the respondent and spouse (when applicable) from the RAND HRS Detailed Imputations File, and we expressed this household income as a percentage of the federal poverty line (FPL) in that year. Although many people with functional impairment live with children or another household member, detailed information about the income these members contribute to the household is not measured in the HRS. In a sensitivity analysis, we followed previous literature in annuitizing non-housing assets^10^ in order to group people by a combined measure of income and assets.

Limitations included possible errors in respondent recall and lack of information about the duration of care in the HRS. We pooled multiple years of data (meaning individuals can be included multiple times) to obtain sufficient sample sizes, so focused on the period prior to the COVID-19 pandemic. However, we acknowledge that COVID-19 may have temporarily and permanently changed population caregiving dynamics. Because we did not include those who receive help only with homemaker tasks (eg, cooking, cleaning) or who pay for care through their residence (eg, assisted living), our findings represent a lower bound on paying for home care out-of-pocket. Sample sizes were somewhat limited, particularly within subgroups. Finally, the HRS does not contain measures of unmet need for care, such as experiencing negative consequences related to lack of assistance, that have been used in other studies and that would allow us to understand the impact of cost-related barriers to accessing home care.^16^

## Results

We estimated that an annual average of approximately 3 million people with personal care needs received home care in 2002–2018, approximately 1 million of whom had dementia. People with dementia were older and had greater functional needs than those without dementia (Appendix Table S2). Among this population, 628 657 people paid out-of-pocket for at least some of this care, approximately half of whom (281 542) had dementia (Figure 1). Fifty-two percent of people who paid out-of-pocket for care (325 603) were poor or “near-poor” (income <100% FPL or between 100% and 200% of the FPL, respectively).

**Figure 2.**
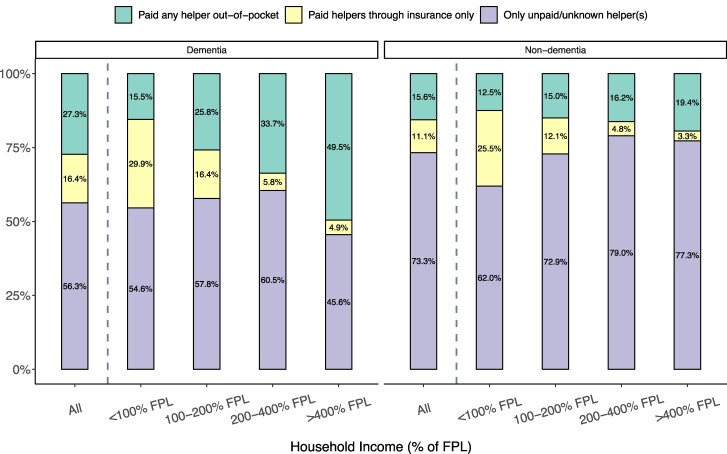
Prevalence of paying out-of-pocket for home care among community-dwelling older adults with personal care needs, by income and dementia status: 2002–2018 Health and Retirement Study (HRS). Based on a sample of 8181 respondent-years from HRS 2002–2018 (2797 dementia, 5384 non-dementia) who received help with at least 1 ADL and did not live in a nursing home or receive ADL help from their residence. Percentages were computed using survey weights. The FPL in 2018 was $12 000 for an individual and $18 000 for a couple. Among all respondents aged 65+ years in our sample years, the income thresholds of 100%, 200%, and 400% FPL corresponded to the 12th, 42nd, and 74th percentiles, respectively. Percentages are calculated using survey weights. Abbreviations: ADL, activities of daily living; FPL, federal poverty line.

Among people with personal care needs who received home care, 27% of people with dementia and 16% of people without dementia paid for care out-of-pocket (Figure 2). The proportion of people paying out-of-pocket increased with income for both groups, but the gradient was significantly more pronounced for people with dementia, rising from 15% in the lowest-income group to 50% in the highest-income group (vs 13% to 19% among those without dementia). Most (80%) of those paying out-of-pocket were paying the full cost of care; the remaining individuals had some care covered by insurance (Appendix Figure S2). Gradients in paying for care out-of-pocket by income remained similar after adjusting for demographic, health and functioning, and family characteristics (Appendix Table S3).

**Figure 3.**
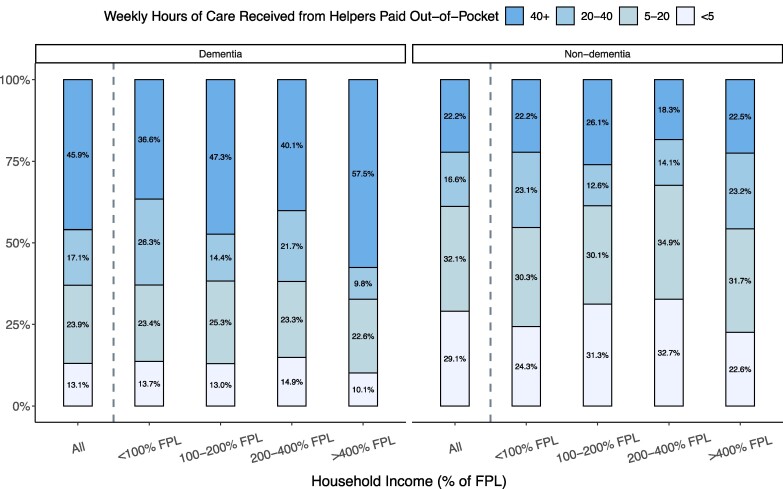
Distribution of average weekly hours of home care received from helpers paid out-of-pocket, among people with any out-of-pocket spending on home care, by income and dementia status: 2002–2018 Health and Retirement Study (HRS). Based on a sample of 967 respondent-years from HRS 2002–2018 (487 dementia, 480 non-dementia) who received help with at least 1 ADL, did not live in a nursing home or receive ADL help from their residence, and reported a nonzero exact out-of-pocket cost amount spent on home care. Percentages were computed using survey weights. The FPL in 2018 was $12 000 for an individual and $18 000 for a couple. Among all respondents aged 65+ years in our sample years, the income thresholds of 100%, 200%, and 400% FPL corresponded to the 12th, 42nd, and 74th percentiles, respectively. Percentages are calculated using survey weights. Abbreviations: ADL, activities of daily living; FPL, federal poverty line.

Among those paying out-of-pocket for care, people with dementia were more likely to pay for full-time help (≥40 h/wk); 46% of people with dementia paid for full-time care vs 22% of people without dementia (Figure 3). For people with dementia, the highest-income group were the most likely to pay out-of-pocket for full-time care (58% of those paying out-of-pocket for home care with dementia and with incomes above 400% FPL paid for full-time help).

**Figure 4.**
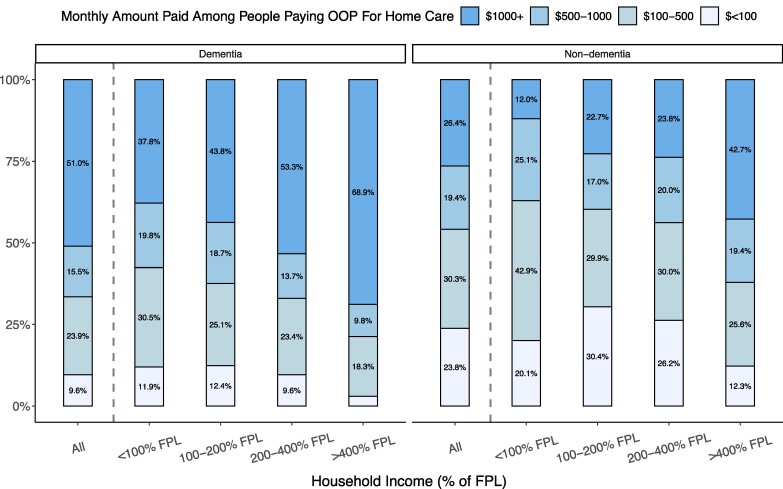
Distribution of monthly out-of-pocket (OOP) spending on home care, among people with any out-of-pocket spending on home care, by income and dementia status: 2002–2018 Health and Retirement Study (HRS). Dollar amounts are expressed in 2018 dollars. Based on a sample of 967 respondent-years from HRS 2002–2018 (487 dementia, 480 non-dementia) who received help with at least 1 ADL, did not live in a nursing home or receive ADL help from their residence, and reported a nonzero exact out-of-pocket cost amount spent on home care. Percentages were computed using survey weights. The FPL in 2018 was $12 000 for an individual and $18 000 for a couple. Among all respondents aged 65+ years in our sample years, the income thresholds of 100%, 200%, and 400% FPL corresponded to the 12th, 42nd, and 74th percentiles, respectively. Percentages are calculated using survey weights. Abbreviations: ADL, activities of daily living; FPL, federal poverty line.

The cost of home care paid out-of-pocket was often high: 51% of people with dementia spent over $1000 per month on home care (2018 dollars) compared to 26% of people without dementia (Figure 4). While care costs increased sharply with income, high costs of care were present even in the lowest-income group, where 38% of people with dementia and 12% of people without dementia spent over $1000 per month on home care.

**Figure 5.**
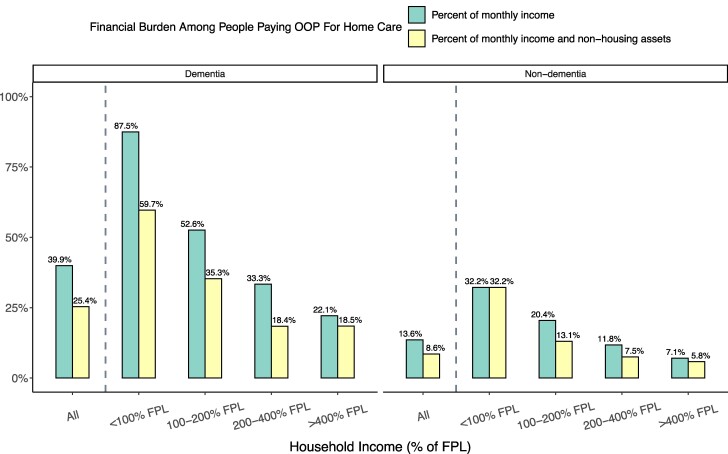
Median financial burden of out-of-pocket (OOP) spending on home care among people with any out-of-pocket spending on home care, by income and dementia status: 2002–2018 Health and Retirement Study (HRS). Financial burden was defined as the ratio of the monthly out-of-pocket spending on home care to a respondent's monthly household income, or monthly income and non-housing assets. Non-housing assets were annuitized following Pearson et al.^10^ Based on a sample of 967 respondent-years from HRS 2002–2018 (487 dementia, 480 non-dementia) who received help with at least 1 ADL, did not live in a nursing home or receive ADL help from their residence, and reported a nonzero exact out-of-pocket cost amount spent on home care. Percentages were computed using survey weights. The FPL in 2018 was $12 000 for an individual and $18 000 for a couple. Among all respondents aged 65+ years in our sample years, the income thresholds of 100%, 200%, and 400% FPL corresponded to the 12th, 42nd, and 74th percentiles, respectively. Percentages are calculated using survey weights. Abbreviations: ADL, activities of daily living; FPL, federal poverty line.

The median person with dementia who paid out-of-pocket for home care spent 40% of their monthly income on home care (vs 14% for the median person without dementia) (Figure 5). Financial burdens are particularly high in the lower-income groups: people who paid out-of-pocket for home care and were poor or near-poor spent 87% and 53% of their income, respectively, on home care. When we incorporated both income and non-housing assets into our measure of financial burden, we found lower, but still substantial, financial burdens of care.

Sensitivity analyses showed that a more stringent definition of paying out-of-pocket for home care lowered total numbers, but patterns by income and dementia status were similar (Appendix Figures S3 and S4). Patterns were also very similar when we grouped respondents by combined income and annuitized wealth (Appendix Figures S5 and S6). Finally, including people who only received help with homemaker tasks (eg, cooking, cleaning) in our sample increased our estimate of the number of people paying for home care out-of-pocket to 755 699 people, but cost and hours estimates remained very similar, as did patterns by income and dementia status (Appendix Figures S7–S11).

## Discussion

More than half a million older adults with personal care needs in the community pay out-of-pocket for home care each year, almost half of whom have dementia. People who pay out-of-pocket for home care span the income spectrum, contradicting a common conception that private-pay home care is used exclusively by high-income or high-wealth individuals. While rates of paying out-of-pocket for home care increased with income, the disproportionate impact of disability and dementia on people of lower socioeconomic status means that approximately half of those who pay out-of-pocket for home care have incomes below 200% FPL.

The large differences in the likelihood of paying out-of-pocket for home care by dementia status may highlight the intensive care needs of people with dementia, who may require 24/7 supervision in addition to help with personal care. Further, our finding of a particularly strong income gradient in the use of private-pay home care among people with dementia (vs those without dementia) suggests that people with dementia with fewer financial resources may face unmet needs. While it is difficult to assess unmet need directly in the HRS, future research should use other data sources to specifically explore unmet care needs among people with dementia across the income spectrum.

Although Medicaid coverage of home- and community-based services has increased dramatically in recent decades, and Medicaid may play an important role in defraying out-of-pocket home care costs for people with limited incomes, we found substantial rates of paying out-of-pocket for home care even among people with incomes below 100% FPL. Almost all states cover older adults with incomes above 74% FPL through Medicaid, and 40% of states cover older adults with incomes below 100% FPL. However, even among people whose incomes qualify them for Medicaid, our results suggest there may remain barriers to accessing Medicaid-funded home care. Such barriers may include strict asset limits or functional eligibility requirements, waitlists for coverage, limits in the number of hours of care people can receive, workforce shortages, and the wide variation across states in coverage of home care.^4-6^ In addition, while policymakers have created alternative Medicaid eligibility pathways (eg, Medically Needy Programs, Special Income Rule) to extend benefits to people with somewhat higher incomes, we found that for all income groups above 100% FPL, people are more likely to pay out-of-pocket for home care than use insurance-funded care. Given these results, understanding barriers to accessing existing benefits among people whose financial resources indicate they are eligible or near-eligible for Medicaid (ie, the “near-poor”) is an important area for future work.

Finally, the financial burden of out-of-pocket payment for home care is significant, particularly among people with dementia and those with limited income and wealth. For some individuals, spending a portion of one's income or savings on home care may reflect an intended use of these funds. However, the long duration of care needs, particularly among people with dementia, may mean that many individuals who pay out-of-pocket may at some point need to decrease their standard of living or rely on financial transfers from family or drawdowns of assets, which could impact generational wealth and family financial well-being. While we do not assess these impacts in this paper, future work could examine the downstream consequences of these out-of-pocket expenses on older adults and their families.

There have been several recent policy proposals to expand protection of older adults against the costs of paying for home care—for example, by including a home care benefit in Medicare.^17-19^ Estimating the costs and benefits of such a program is challenging because little is known about how many people would take-up such a program; some people may still prefer to receive care in a residential setting or from unpaid family members. Our findings suggest that such a benefit would indeed come at a substantial cost to the government, given that it is likely that the 600 000 individuals currently paying out-of-pocket for home care would use such a benefit if available, and additional users who currently do not pay privately for care may take-up a program that covers some of the costs of this care. However, we also found that more than half of the individuals currently paying out-of-pocket for home care have incomes below 200% FPL, and may thus be likely to be at risk of unmet need or financial fragility as a result of this spending.

## Conclusion

While paying out-of-pocket for home care is more common with increasing income, a substantial minority of low-income older adults—particularly those with dementia—pay out-of-pocket for home care. Policymakers could consider ways to better protect lower-income groups from the financial risk of paying out-of-pocket for home care. Potential policy solutions include expanding access to Medicaid home- and community-based services, policy supports to encourage saving for long-term care, development of broader-based long-term-care insurance programs, and expansion of Medicare to cover home care. Given the substantial costs of such programs, efforts should also be made to design policies that are targeted toward the most vulnerable individuals, and that include incentives (eg, cost-sharing) that encourage responsible use and individual contributions toward costs of care for those who have the means to do so.

## Supplementary Material

qxae180_Supplementary_Data
