# Supplementary material for: Paying for home care out-of-pocket is common and costly across the income spectrum among older adults
Source: Health Aff Sch. 2025 Jan 16;3(1):qxae180. doi: 10.1093/haschl/qxae180 (PMC11736716; doi:10.1093/haschl/qxae180)
Supplement: qxae180_Supplementary_Data [file qxae180_supplementary_data.zip › SelfFundedCare_Appendix_Final_HAS_Revised.docx]

Supplemental Appendix for “Paying for home care out-of-pocket is common and costly across the income spectrum among older adults with dementia”

**Appendix Table 1: Sample selection**

|  | Total | PLWD | Non-PLWD |
| --- | --- | --- | --- |
| Age > 65 | 97919 | 10282 | 87637 |
| Age > 65 and received help with 1 or more ADLs | 11368 | 5173 | 6125 |
| Nursing home resident | 3023 | 2389 | 634 |
| Received ADL help from residence | 164 | 57 | 107 |
| Non-NH resident and did not receive ADL help from residence | **8181** | **2797** | **5384** |

Notes: Sample is constructed at the respondent-year level, using 9 waves of data (2002-2018).

**Appendix Figure 1: Identifying helpers paid out-of-pocket**

**
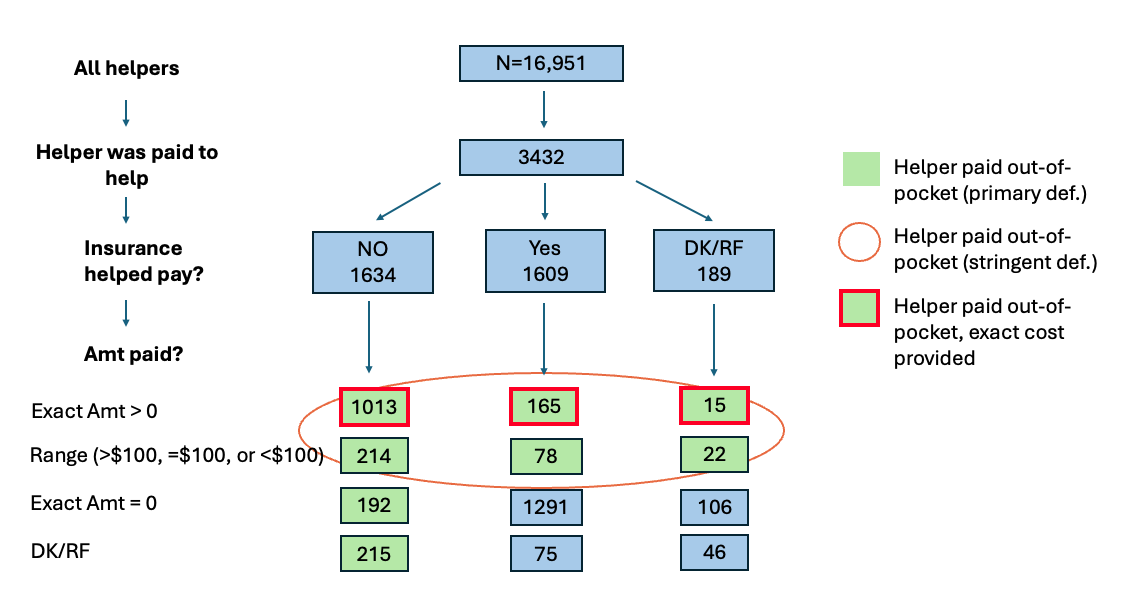
**

Notes: Exhibit shows how the questions in the HRS that we use to define whether respondents paid out-of-pocket for home care. For all helpers, respondents were first asked if the helper was paid to help, then if insurance helped pay for the helper, and then finally the amount the respondent or their spouse paid out-of-pocket for that helper in the last month. In cases where the respondent does not know or refuses to answer the question about an exact out-of-pocket cost (19% of cases), the survey then asks them whether they paid more, about, or less than $100 for that helper. Respondents provided one of these ranges in 47% of cases where they did not provide an exact out of pocket cost.

Provided sample sizes are unique helpers in our sample. The green shaded boxes are all included in our primary definition of helpers paid out-of-pocket. Boxes inside the orange circle are included in our more stringent definition. Finally, boxes outlined in red are used in Exhibits 3-6 in the paper to describe characteristics (hours, cost, financial burden) of people who paid for home care out-of-pocket, where we require that the respondent provided an exact nonzero out-of-pocket cost.

Of note, while the question about out-of-pocket costs of care asks about the amount the “respondent or their spouse” paid for a helper, a follow-up question asks whether someone else (e.g. a child) helped pay that cost (respondents answered “Yes” to this follow-up question for 9% of the helpers that we identify as being paid for out-of-pocket in our sample). We thus interpret our definition of out-of-pocket payments for home care as capturing helpers that were paid by the respondent, their spouse, and/or other family or friends.

**Appendix Table 2: Descriptive statistics of individuals living at home with personal care needs, by dementia status and income**

|  | Dementia | | | | | Non-dementia | | | | |  |
| --- | --- | --- | --- | --- | --- | --- | --- | --- | --- | --- | --- |
|  | Overall | <100% | 100-200% | 200-400% | >400% | Overall | <100% | 100-200% | 200-400% | >400% | |
| Observations | 2797 | 802 | 1141 | 603 | 251 | 5384 | 1146 | 1965 | 1490 | 783 | |
| **Demographic** |  |  |  |  |  |  |  |  |  |  | |
| Age | 83.03 | 82.85 | 83.34 | 82.67 | 83.08 | 77.35 | 77.21 | 78.09 | 76.94 | 76.62 | |
| Male | 34.77% | 26.82% | 33.09% | 40.94% | 48.18% | 37.23% | 22.05% | 32.03% | 42.77% | 56.38% | |
| Race |  |  |  |  |  |  |  |  |  |  | |
| White | 74.69% | 59.44% | 74.36% | 84.33% | 93.64% | 81.48% | 63.38% | 81.85% | 88.65% | 88.72% | |
| Black | 18.89% | 31.15% | 18.71% | 11.31% | 5.05% | 12.92% | 23.16% | 13.90% | 8.87% | 6.20% | |
| Other | 6.42% | 9.41% | 6.93% | 4.36% | 1.31% | 5.60% | 13.47% | 4.25% | 2.48% | 5.09% | |
| Hispanic | 17.36% | 30.53% | 17.68% | 7.66% | 3.93% | 11.13% | 30.10% | 9.67% | 5.60% | 2.39% | |
| Census Region |  |  |  |  |  |  |  |  |  |  | |
| Northeast | 17.14% | 14.28% | 19.78% | 16.03% | 16.33% | 17.36% | 15.81% | 19.35% | 16.87% | 15.67% | |
| Midwest | 19.36% | 12.52% | 19.52% | 28.31% | 16.10% | 22.18% | 16.42% | 22.12% | 26.12% | 21.91% | |
| South | 45.85% | 59.29% | 42.31% | 38.88% | 41.42% | 40.60% | 51.98% | 40.29% | 36.24% | 35.99% | |
| West | 17.55% | 13.56% | 18.39% | 16.78% | 25.93% | 19.67% | 15.14% | 18.06% | 20.74% | 26.42% | |
| **Health and Functioning** |  |  |  |  |  |  |  |  |  |  | |
| # ADL difficulties |  |  |  |  |  |  |  |  |  |  | |
| 1 | 14.95% | 11.65% | 15.91% | 16.04% | 17.18% | 28.53% | 22.62% | 26.27% | 33.23% | 31.95% | |
| 2 | 15.76% | 16.75% | 16.33% | 15.86% | 10.70% | 24.11% | 23.81% | 23.82% | 23.71% | 25.81% | |
| 3 | 16.96% | 18.36% | 16.43% | 17.70% | 13.90% | 19.15% | 19.47% | 19.26% | 17.80% | 20.95% | |
| 4 | 14.42% | 15.29% | 14.88% | 13.89% | 11.51% | 12.69% | 11.49% | 13.97% | 12.97% | 10.76% | |
| 5 | 15.27% | 16.56% | 13.75% | 14.96% | 18.75% | 9.89% | 15.38% | 9.87% | 8.42% | 6.20% | |
| 6 | 22.64% | 21.39% | 22.72% | 21.55% | 27.97% | 5.63% | 7.22% | 6.81% | 3.88% | 4.32% | |
| Years since first ADL | 3.57 | 4.85 | 3.55 | 2.43 | 2.93 | 3.91 | 4.63 | 3.93 | 3.60 | 3.59 | |
| # IADL difficulties |  |  |  |  |  |  |  |  |  |  | |
| 0 | 5.01% | 6.80% | 5.50% | 3.54% | 1.73% | 31.95% | 24.03% | 27.89% | 36.04% | 42.77% | |
| 1 | 6.30% | 7.91% | 5.86% | 6.74% | 2.93% | 24.86% | 24.19% | 25.35% | 25.21% | 23.94% | |
| 2 | 10.41% | 10.27% | 11.85% | 9.81% | 6.35% | 23.04% | 24.76% | 24.16% | 22.22% | 20.04% | |
| 3 | 13.71% | 15.52% | 14.14% | 11.03% | 13.32% | 11.38% | 15.77% | 12.17% | 9.55% | 7.81% | |
| 4 | 24.82% | 25.19% | 21.72% | 28.45% | 28.24% | 6.14% | 7.74% | 7.34% | 5.03% | 3.59% | |
| 5 | 39.75% | 34.31% | 40.93% | 40.42% | 47.42% | 2.64% | 3.50% | 3.10% | 1.96% | 1.85% | |
| Chronic Conditions |  |  |  |  |  |  |  |  |  |  | |
| High Blood Pressure | 72.20% | 77.83% | 72.44% | 69.66% | 62.48% | 77.51% | 84.07% | 77.82% | 74.83% | 74.00% | |
| Diabetes | 33.50% | 42.21% | 31.16% | 33.21% | 21.30% | 39.71% | 47.06% | 39.34% | 38.85% | 33.56% | |
| Cancer | 20.90% | 18.91% | 20.38% | 21.93% | 25.81% | 24.29% | 18.58% | 23.73% | 26.02% | 29.01% | |
| Lung Disease | 14.27% | 16.78% | 13.26% | 14.67% | 11.04% | 23.28% | 27.56% | 27.03% | 18.15% | 19.19% | |
| Heart Disease | 44.06% | 42.55% | 45.21% | 45.28% | 40.63% | 48.89% | 45.16% | 49.32% | 49.76% | 50.69% | |
| Stroke | 33.09% | 27.41% | 32.67% | 38.01% | 38.42% | 21.19% | 20.67% | 22.23% | 22.14% | 17.76% | |
| Psychiatric | 28.98% | 32.98% | 27.71% | 26.74% | 28.79% | 31.48% | 37.38% | 31.25% | 30.62% | 26.71% | |
| Arthritis | 70.37% | 75.98% | 68.97% | 69.39% | 63.83% | 86.30% | 89.48% | 86.05% | 84.41% | 86.57% | |
| **Family** |  |  |  |  |  |  |  |  |  |  | |
| Household Type |  |  |  |  |  |  |  |  |  |  | |
| Lives alone | 18.91% | 25.67% | 18.68% | 14.57% | 12.79% | 22.21% | 32.73% | 27.26% | 15.33% | 11.85% | |
| Partner-only | 31.15% | 17.93% | 27.38% | 42.58% | 53.38% | 43.99% | 20.03% | 35.06% | 58.49% | 64.09% | |
| Partner and at least one other | 12.32% | 10.64% | 12.51% | 14.04% | 11.90% | 12.56% | 10.21% | 11.44% | 13.86% | 15.27% | |
| Child, no partner | 30.69% | 36.28% | 34.38% | 24.17% | 16.74% | 17.65% | 31.07% | 22.32% | 9.98% | 6.35% | |
| Other | 6.93% | 9.48% | 7.05% | 4.64% | 5.18% | 3.58% | 5.96% | 3.92% | 2.34% | 2.44% | |
| Any Child Lives Within 10 Miles | 62.63% | 64.05% | 65.67% | 59.03% | 55.19% | 61.54% | 65.23% | 62.40% | 63.55% | 51.91% | |
| **Insurance** |  |  |  |  |  |  |  |  |  |  | |
| Enrolled in Medicaid | 29.16% | 59.75% | 28.96% | 6.98% | 2.58% | 19.40% | 54.79% | 20.21% | 5.67% | 2.29% | |
| Covered by Long-Term Care Insurance | 9.24% | 6.84% | 6.64% | 11.53% | 20.32% | 10.86% | 4.71% | 8.51% | 12.19% | 20.59% | |
| **Potential Markers of Financial Vulnerability** |  |  |  |  |  |  |  |  |  |  | |
| Did not always have enough money to buy food | 9.85% | 17.32% | 8.87% | 3.08% | 1.06% | 13.32% | 24.26% | 17.29% | 5.00% | 0.94% | |
| Received Financial Help of $500 or more from children/grandchildren | 17.57% | 14.32% | 20.50% | 16.70% | 15.69% | 12.68% | 15.13% | 15.14% | 10.30% | 6.62% | |
| ^1^Mean; % | | | | | | | | | | |  |

**Appendix Figure 2: Prevalence of paying entirely out-of-pocket for home care and paying for home care with both insurance and out-of-pocket payments among community-dwelling older adults with personal care needs, by income and dementia status,**

Notes: See Exhibit 2 in the main text. Respondents that paid helpers entirely out-of-pocket indicated that insurance did not help pay for any paid helper, while respondents that paid helpers out-of-pocket and with insurance indicated that insurance helped pay, but also reported a nonzero out-of-pocket payment for home care.

**Appendix Table 3: Unadjusted and adjusted estimates of probability of paying for home care out-of-pocket**

1. Predicted probabilities of paying out-of-pocket for home care by income, unadjusted and adjusting for demographic, health and family characteristics

|  | **Dementia** | | **Non-Dementia** | |
| --- | --- | --- | --- | --- |
|  | **Unadjusted** | **Adjusted** | **Unadjusted** | **Adjusted** |
| **<100% FPL** | 0.148 (0.014) | 0.152 (0.016) | 0.121 (0.013) | 0.078 (0.014) |
| **100-200% FPL** | 0.247 (0.015) | 0.242 (0.014) | 0.143 (0.010) | 0.122 (0.009) |
| **200-400% FPL** | 0.330 (0.023) | 0.339 (0.020) | 0.159 (0.011) | 0.188 (0.011) |
| **>400% FPL** | 0.472 (0.035) | 0.463 (0.032) | 0.189 (0.016) | 0.234 (0.015) |
| **N** | 2797 | 2797 | 5384 | 5384 |

Notes: Standard errors in parentheses. Unadjusted estimates are predictions from an ordinary least square regression of paying out of pocket for home care on income, while adjusted estimates are from regression including demographic, health, and family characteristics (regression estimates shown before).

1. Regression coefficient estimates from estimate of relationship between paying out-of-pocket for home care and demographic, health, and family characteristics

|  | Dementia |  | Non-dementia |  |
| --- | --- | --- | --- | --- |
| **Income** | Coefficient | SE | Coefficient | SE |
| <100% FPL | 0.000 | (.) | 0.000 | (.) |
| 100-200% FPL | 0.090^***^ | (0.021) | 0.043^*^ | (0.017) |
| 200-400% FPL | 0.187^***^ | (0.027) | 0.110^***^ | (0.020) |
| >400% FPL | 0.310^***^ | (0.037) | 0.155^***^ | (0.023) |
| Age | 0.005^***^ | (0.001) | 0.004^***^ | (0.001) |
| Male | -0.037 | (0.021) | 0.001 | (0.014) |
| **Race/Ethnicity** |  |  |  |  |
| White / Caucasian | 0.000 | (.) | 0.000 | (.) |
| Black / African American | -0.078^***^ | (0.019) | -0.027 | (0.016) |
| Other | -0.082^*^ | (0.039) | -0.000 | (0.027) |
| Hispanic | 0.000 | (0.000) | 0.000 | (0.000) |
| **# ADL difficulties** |  |  |  |  |
| 1 | 0.000 | (.) | 0.000 | (.) |
| 2 | 0.077^**^ | (0.028) | -0.022 | (0.014) |
| 3 | 0.128^***^ | (0.029) | 0.034^*^ | (0.017) |
| 4 | 0.120^***^ | (0.031) | 0.026 | (0.020) |
| 5 | 0.117^***^ | (0.031) | 0.088^***^ | (0.023) |
| 6 | 0.228^***^ | (0.031) | 0.103^**^ | (0.033) |
| **# IADL difficulties** |  |  |  |  |
| 0 | 0.000 | (.) | 0.000 | (.) |
| 1 | 0.011 | (0.045) | 0.035^*^ | (0.014) |
| 2 | 0.011 | (0.043) | 0.095^***^ | (0.016) |
| 3 | 0.026 | (0.042) | 0.099^***^ | (0.023) |
| 4 | 0.040 | (0.040) | 0.112^***^ | (0.031) |
| 5 | 0.052 | (0.040) | 0.099^*^ | (0.040) |
| Years since first ADL | -0.003 | (0.002) | -0.003^**^ | (0.001) |
| High blood pressure | -0.032 | (0.022) | -0.007 | (0.014) |
| Diabetes | -0.030 | (0.019) | -0.007 | (0.012) |
| Cancer | -0.014 | (0.022) | 0.011 | (0.014) |
| Lung disease | 0.009 | (0.025) | 0.005 | (0.014) |
| Heart disease | -0.008 | (0.018) | -0.012 | (0.011) |
| Stroke | -0.001 | (0.019) | 0.024 | (0.014) |
| Psychiatric | -0.008 | (0.020) | -0.017 | (0.013) |
| Arthritis | -0.057^**^ | (0.021) | -0.003 | (0.016) |
| **Household type** |  |  |  |  |
| Lives alone | 0.000 | (.) | 0.000 | (.) |
| Partner-only | -0.180^***^ | (0.031) | -0.196^***^ | (0.020) |
| Partner and at least one other | -0.199^***^ | (0.035) | -0.206^***^ | (0.022) |
| Child, no partner | -0.098^***^ | (0.029) | -0.104^***^ | (0.021) |
| No partner or children, but not alone | 0.003 | (0.040) | -0.047 | (0.037) |
| Child lives within 10 mi | -0.000^*^ | (0.000) | -0.000 | (0.000) |
| **Census Region** |  |  |  |  |
| Northeast | 0.000 | (.) | 0.000 | (.) |
| Midwest | -0.014 | (0.030) | 0.048^**^ | (0.018) |
| South | -0.046 | (0.025) | 0.018 | (0.015) |
| West | 0.063 | (0.033) | 0.086^***^ | (0.020) |
| Other | -0.131 | (0.115) | 0.320 | (0.177) |
| Constant | -0.161 | (0.125) | -0.219^**^ | (0.075) |
| R-squared | 0.160 |  | 0.135 |  |
| Number of observations | 2797 |  | 5384 |  |

Notes: ^*^ *p* < 0.05, ^**^ *p* < 0.01, ^***^ *p* < 0.001 Standard errors in parentheses. Unadjusted estimates are predictions from an ordinary least square regression of paying out of pocket for home care on income, while adjusted estimates are from regression including demographic, health, and family characteristics (regression estimates shown before).

**Appendix Figure 3: Annual average number community-dwelling older adults with personal care needs receiving home care from different sources by income and dementia status, using more stringent definition of paying out-of-pocket for home care**

Notes: See Exhibit 1 in the main text and Appendix Exhibit 2. The more stringent definition of paying out-of-pocket for home care excludes people who reported having paid helpers that were not paid by insurance, but also reported having zero or unknown out-of-pocket cost.

**Appendix Exhibit 4: Prevalence of paying out-of-pocket for home care among community-dwelling older adults with personal care needs, by income and dementia status, using more stringent definition of paying out-of-pocket for home care**

Notes: See Exhibit 2 in the main text and Appendix Exhibit 2. The more stringent definition of paying out-of-pocket for home care excludes people who reported having paid helpers that were not paid by insurance, but also reported having zero or unknown out-of-pocket cost.

**Appendix Figure 5: Annual average number of community-dwelling older adults with personal care needs receiving home care from different sources, by combined income and assets and dementia status**

Notes: See Figure 1 in the main text. Non-housing assets were annuitized following Pearson, et al. (2019), and then added to annual income and grouped by percentiles to match Exhibit 1.

**Appendix Figure 6: Annual average number of people receiving help of different types each year, among people living at home who received help with at least one ADL, grouping by percentiles of income and annuitized assets**

Notes: See Figure 2 in the main text. Non-housing assets were annuitized following Pearson, et al. (2019), and then added to annual income and grouped by percentiles to match Exhibit 2.

**Appendix Figure 7: Annual average number of community-dwelling older adults with personal care or homemaker needs receiving home care from different sources, by income and dementia status**

Notes: See Figure 1 in the main text.

**Appendix Figure 8: Prevalence of paying out-of-pocket for home care among community-dwelling older adults with personal care or homemaker needs, by income and dementia status**

Notes: See Figure 2 in the main text.

**Appendix Figure 9: Distribution of average weekly hours of home care received from helpers paid out-of-pocket, among people with any out-of-pocket spending on home care, by income and dementia status, sample with personal care or homemaker needs**

Notes: See Figure 3 in the main text.

**Appendix Figure 10: Distribution of monthly out-of-pocket spending on home care, among people with any out-of-pocket spending on home care, by income and dementia status, sample with personal care or homemaker needs**

Notes: See Figure 4 in the main text.

**Appendix Figure 11: Median financial burden of out-of-pocket spending on home care among people with any out-of-pocket spending on home care, by income and dementia status**

Notes: See Figure 5 in the main text.
